# Cell Type-Dependent RNA Recombination Frequency in the Japanese Encephalitis Virus

**DOI:** 10.1155/2014/471323

**Published:** 2014-07-22

**Authors:** Wei-Wei Chiang, Ching-Kai Chuang, Mei Chao, Wei-June Chen

**Affiliations:** ^1^Division of Microbiology, Graduate Institute of Biomedical Sciences, Chang Gung University, Kwei-San, Tao-Yuan 33332, Taiwan; ^2^Department of Plant Pathology, University of Kentucky, Lexington, KY 40546-0312, USA; ^3^Department of Microbiology and Immunology, Chang Gung University, Kwei-San, Tao-Yuan 33332, Taiwan; ^4^Department of Public Health and Parasitology, Chang Gung University, Kwei-San, Tao-Yuan 33332, Taiwan

## Abstract

Japanese encephalitis virus (JEV) is one of approximately 70 flaviviruses, frequently causing symptoms involving the central nervous system. Mutations of its genomic RNA frequently occur during viral replication, which is believed to be a force contributing to viral evolution. Nevertheless, accumulating evidences show that some JEV strains may have actually arisen from RNA recombination between genetically different populations of the virus. We have demonstrated that RNA recombination in JEV occurs unequally in different cell types. In the present study, viral RNA fragments transfected into as well as viral RNAs synthesized in mosquito cells were shown not to be stable, especially in the early phase of infection possibly via cleavage by exoribonuclease. Such cleaved small RNA fragments may be further degraded through an RNA interference pathway triggered by viral double-stranded RNA during replication in mosquito cells, resulting in a lower frequency of RNA recombination in mosquito cells compared to that which occurs in mammalian cells. In fact, adjustment of viral RNA to an appropriately lower level in mosquito cells prevents overgrowth of the virus and is beneficial for cells to survive the infection. Our findings may also account for the slower evolution of arboviruses as reported previously.

## 1. Introduction

Japanese encephalitis (JE) is an important mosquito-borne viral disease, occasionally causing encephalitic symptoms [1]. Nowadays, it is extensively distributed in most Asian countries and was also recently reported from Australia [2]. The JE virus (JEV) is one of some 70 members of the genus* Flavivirus* belonging to the family Flaviviridae [3], the genome of which contains a linear, single-stranded positive-sense RNA (~11 kb long) that encodes 3 structural proteins including nucleocapsid (C), membrane (preM/M), and envelope (E) proteins, as well as 7 nonstructural proteins (NS1, NS2A, NS2B, NS3, NS4A, NS4B, and NS5) [4]. Due to lack of a proofreading mechanism and an inability to repair errors during RNA synthesis, spontaneous mutations frequently occur which contribute to the formation of genetically diversified populations or so-called “quasispecies” in flaviviruses including the JEV [5].

Maintenance of genetic diversity theoretically reduces the rapid loss of fitness via Muller's ratcheting during viral passage from one host to another [6], which provides benefits to a virus that is adapting to a new niche or selective regimen of its environment [7]. Possibly, this feature differentially occurs in different types of host cells [8]. In addition to gene mutations [9], RNA recombination, at least in some cases, can also serve as a factor helping a virus escape from accumulated deleterious effects in a viral population [10]. In other words, RNA recombination may serve as an alternate means to generate genetic changes [11] and likely produces a new form of RNA comprising genetic information from multiple sources [12].

The viral RNA recombination was first reported in the poliovirus, a picornavirus [13], and subsequently in a variety of viruses that infect humans, animals, plants, and bacteria [14–18]. Therefore, a new virus may be generated through RNA recombination between different strains. Among arboviruses, at least the western equine encephalitis virus is believed to be a recombinant virus that arose from distant viral progenitors, including an eastern equine encephalitis virus-like virus and a Sindbis-like virus [19]. As a result, the ability to form unpredictable recombinant strains or species between virus populations is of considerable concern [20], particularly the possibility of RNA recombination occurring from cocirculated live-attenuated vaccine strains and wild viruses during synthesis of new RNAs [21, 22].

Flaviviruses naturally comprise multiple genotypes or strains [23, 24], making them likely to undergo RNA recombination. The first RNA recombination of the JEV was proposed based on a bioinformatics analysis [17]. Furthermore, RNA recombination was found to occur unequally in mosquito and mammalian cells [25]. Herein, we provided evidences of RNA recombination of the JEV that occurs at a lower frequency in mosquito cells, which may, at least partly, contribute to evolution of the virus [26].

## 2. Materials and Methods

### 2.1. Viruses and Cell Lines

Three strains of the JEV, including Nakayama (the vaccine strain), T1P1-S1 (a small plaque clone from the T1P1 strain) [27], and CJN-S1 (a small plaque clone from the CJN strain, a kind gift from Dr. M. H. Ho, Academia Sinica, Taipei, Taiwan), were used in this study. Of these, further purification via the plaque-picking method to select T1P1-S1 and CJN-S1 strains was implemented as part of the present study [27]. The viruses were propagated in C6/36 mosquito cells and titrated in baby hamster kidney- (BHK-) 21 cells. Both cell lines were maintained as previously described [27].

### 2.2. Virus Titration

Virus titers were determined by means of a plaque assay of BHK-21 cells following descriptions in our previous report [27]. Calculation of virus titers was based on the number of formed plaques, expressed as plaque-forming units (pfu)/mL.

### 2.3. Reverse Transcriptase-Polymerase Chain Reaction (RT-PCR)

To detect viral infection in cells, extracted RNA was applied to perform RT with the reverse primer at 42°C for 30 min to generate complementary (c) DNA. PCR cycling was then carried out using the forward primer which was subsequently run to amplify a gene fragment with a size of 529 bp under the following conditions: 25 cycles of 95°C for 30 s, 60°C for 30 s, and 72°C for 1 min. The primers used to amplify specific regions are presented in individual sections below. All procedures in this portion of the study followed our previous description [25].

### 2.4. Assay for Coinfection and RNA Recombination of Viral Strains

Coinfection of JEV strains was verified by a method described in our previous report [25]. In brief, extracted viral RNAs were applied to perform the RT-PCR with the primer pair, 10-36F (5′-CTGTGTGAACTTCTTGGCTTAGTATCG-3′) and 850-877R (5′-CAGTTTTCATGAGATATCGTGTGTGGC-3′). Fragments (868 bp) amplified from JEV strains simultaneously infecting BHK-21 or C6/36 cells were subjected to restriction fragment length polymorphism (RFLP) with the restriction enzyme RsaI to verify coinfection. A pattern showing fragments of 219, 401, and 248 bp represented T1P1-S1 infection, while that showing fragments of 219 and 649 bp represented CJN-S1 infection. Those exhibiting all size of fragments indicated that both viral strains had coinfected a single cell. In addition, RFLP using specific restriction enzymes as shown in our previous report [25] was used to verify RNA recombination between viral strains in a single cell. In some experiments for assay of RNA recombination, RFLP was carried out by using cells cultured in the presence of an exoribonuclease inhibitor (3′-phosphoadenosine-5′-phosphate, PAP) (Sigma-Aldrich, St. Louis, MO, USA).

### 2.5. Construction of the Plasmid p(+)T1P1-5′3′-Untranslated Region- (UTR-) I

In order to evaluate RNA recombination between genomic RNA and a transfected RNA sequence, the p(+)T1P1-5′3′-UTR-1 plasmid was constructed as described here. Viral RNA derived from the T1P1 strain of the JEV was used as a template to generate DNA fragments corresponding to the 5′- or 3′-end of genomic-sense RNA. To prepare the 5′-end sequence, a primer (5′-CTGCCAAGCATCCAGCCAAGTA-3′, complementary to nt 895~916 of the 5′-end of the T1P1 genome) was used for RT to synthesize the first-strand cDNA. Subsequently, another primer (5′-TAATACGACTCACTATAGAGAAGTTTATCTGTGTG-3′) containing a partial sequence of the T7 polymerase promoter used as a tag (italicized) at the 5′-end (nt 1~18) of the T1P1 5′-end sequence was used in the PCR to amplify a 934 bp DNA fragment. In the meantime, the primer 5′-GTGTTCTTCCTCACCACCAGCTAC-3′ (nt 10,946~10,969 at the 3′-end of the T1P1 genome) was used for RT to generate cDNA. Another primer (5′-GAAAATTATGTTGACTAC-3′, corresponding to the sequence nt 10,320~10,337) was subsequently used for the PCR under conditions described above to amplify a 650 bp DNA fragment. Both types of PCR products were separately digested with the restriction enzyme, AatII; the resultant DNA fragments were ligated to form subgenomic DNA which contained both 5′-end (nt 1~599) and 3′-end (nt 10,367~10,969) sequences. Subsequently, the subgenomic DNAs were cloned into pGEM-T (Promega, Madison, WI, USA) to form a plasmid designated p(+)T1P1-5′3′-UTR-I which contained an insert of a 1202 bp fragment.

### 2.6. Construction of the p(+)5′3′-UTR-II Plasmid

In order to see the stability of viral fragments in host cells, the p(+)5′3′-UTR-II plasmid was constructed. To construct the plasmid, the pT1P1-5′3′-UTR was used as a template, and the PCR was performed under conditions described above with the primers 5′-TAATACGACTCACTATAGAGAAGTTTATCTGTGTG-3′ (the italics indicate a partial T7 polymerase promoter sequence) and 5′-AAGATATCGTGTTCTTCCTCAC CACC-3′ (the italics indicate an EcoRV restriction enzyme site). The PCR products were digested with SpeI and AatII to delete a fragment from nt 178~599; the resultant DNA fragments were then treated with Klenow Fragment enzyme (Fermentas, Hanover, MD, USA) and ligated to form subgenomic DNA which only contained the 5′- and 3′-UTRs of the T1P1 genome. Subsequently, the subgenomic DNAs were cloned into pGEM-T (Promega, Fitchburg, WI, USA) to form plasmids designated p(+)5′3′-UTR-II.

### 2.7. Preparation of the Positive (+) and Negative (−) Sense 5′-End RNA Sequences and Derived dsRNA

Both (+) and (−) sense 5′-end RNA sequences were prepared from the pT1P1-5′3′-UTR-II plasmid. In preparation of the (+) sense 5′-end RNA sequence, the plasmid was linearized by NdeI and transcribed with T7 RNA polymerase using an in vitro transcription system (Fermentas). The RNA products (599 bp) were extracted with phenol-chloroform, precipitated in ethanol, and then stored in a deep freezer until used for transfection. To prepare the (−) sense 5′-end RNA sequence, the plasmid was first linearized, and the 3′-end sequence of the subgenomic DNA was deleted with NdeI. The resultant linear forms of the plasmid were religated and then redigested with SacII. The products were transcribed with T7 RNA polymerase using an in vitro transcription system (Fermentas) to generate the (−) sense 5′-end RNA sequence which was harvested as done for the (+) sense 5′-end RNA sequence. To prepare dsRNA, positive- and negative-stranded RNA described from pT1P1-5′3′-UTR-II were mixed together, incubated at 95°C for 5 min and then 4°C for 10 min. Ultimately, 2 *μ*L RNase was added to cleave single-stranded RNA that failed to anneal in the mixture. The product was used to evaluate degradation of dsRNA fragments in host cells.

### 2.8. Transfection of dsRNA or the (+) sense 5′-end RNA Sequence and Viral Infection in Cells

Transfection of dsRNA or (+) sense 5′-end RNA-I prepared from the plasmids (+) pT1P1-5′3′-UTR-II was carried out in BHK-21 and C6/36 cells. At 5 h posttransfection (hpt), cells were infected with the Nakayama strain of the JEV, at an MOI of 5. The detailed procedure followed a previous description, from which efficacy of transfection was demonstrated [25].

### 2.9. Assessment of RNA Stability by an RT-PCR

Sequences derived from (+)5′3′-UTR-II RNA were transfected into cells either treated or untreated with an exoribonuclease inhibitor (3′-phosphoadenosine-5′-phosphate, PAP) (Sigma-Aldrich, St. Louis, MO, USA) and incubated for 5 h. RNA was extracted with the TRIzol reagent (5 PRIME, Gaithersburg, MD, USA), and then DNAse (Promega) was added to delete interference of genomic DNA. RT was subsequently run in a mixture containing 4 *μ*g RNA, 1 *μ*L 100 mM random hexamer primer, and 1 *μ*L 10 mM dNTP, and double-distilled (dd) H_2_O water was added to bring the volume up to 12 *μ*L. This was heated at 65°C for 5 min, allowed to stand at 4°C for 2 min, and then 4 *μ*L 5x first-strand buffer, 2 *μ*L dithiothreitol (DTT), 1 *μ*L of an RNase inhibitor (RNase OUTTM; Invitrogen, Carlsbad, CA, USA) were added. After incubation at room temperature for 5 min, 1 *μ*L of reverse transcriptase M-MLV (Invitrogen) was added and allowed to react for 1 h at 37°C, followed by 15 min at 75°C. The cDNA produced was then used for the subsequent PCR under the conditions described above. The primers used for the PCR included the 5′-UTR (5′-AGAAGTTTATCTGTGTGAAC-3′) and 3′-UTR (5′-AGATCCTGTGTTCTTCC-3), which generated PCR products predicted to be 907 bp. To assess integration of dsRNA, the same cDNA and primer pair described above were used to amplify a fragment 807 bp long.

### 2.10. Assay for RNA Recombination from Transfected as well as Infected Cells

BHK-21 and C6/36 cells transfected with transcribed RNA fragments were then infected by the Nakayama strain of the JEV. Total RNA extracted from cells that had been transfected with (+) sense 5′-end RNA-1 was run for RT using a primer (850-877R: 5′-TCAGTTTTCATGAGATATCGTGTGTGGC-3′) complementary to the sequence of nt 850~877. Amplification using the forward primer (RVF1: 5′-GCGGGATTTAATACGACTCACTATAG-3′) which is a partial sequence of the plasmid that serves as a tag and the reverse primer (RVR1/nt 516~538: 5′-CTGCAATATCCGTATTGTTGAC-3′) produced a specific region comprised of 564 nt. The reverse primer used here was specific for the Nakayama strain. As a result, the fragments amplified by this primer pair must represent a strain of genetic recombination.

### 2.11. Measurement of Viral RNA Accumulated in Cells Infected by the JEV

Viral replication was validated by RNA accumulation through a real-time RT-PCR with cDNAs reverse-transcribed from extracted RNA of infected (at an MOI of 1) or uninfected C6/36 and BHK-21 cells. The primer pairs TS1-F/TS1R (5′-TGTGGCTTGCGAGCTTGGCAG-3′/5′-ACATGTAGCCGACGTCGATT-3′) and CJN1-F/CJN1R (5′-TGTGGCTTGCGAGCTTGGCTA-3′/5′-ACATGTAGCCGACGTCTATC-3′) were used to amplify specific regions of the T1P1-S1 and CJN-S1 strains, respectively. Levels of 18S rRNA designed from the genome of C6/36 or BHK-21 cells were also amplified as an internal control as our previous report [25]. Results are expressed as the relative quantities, so fold change was used to represent the amount of viral RNA that accumulated at each time point of infection. To monitor synthesis of viral RNA including positive and negative strands in a time course in C6/36 cells, viral RNA extracted from infected cells (0~15 hpi) was used to run RT-PCR as the procedures described previously [27]. As above, 18S rRNA designed from the genome of C6/36 cells was also amplified as an internal control. The amplified cDNA fragment was then identified by running the PCR product on a 2% (w/v) agarose gel.

### 2.12. Statistical Analysis

Yates' chi-square test was used to assess the frequency of RNA recombination in cells coinfected by two virus strains or transfected by viral RNA fragments.

## 3. Results

### 3.1. RNA Recombination in BHK-21 Cells and C6/36 Cells

**Figure 1:**
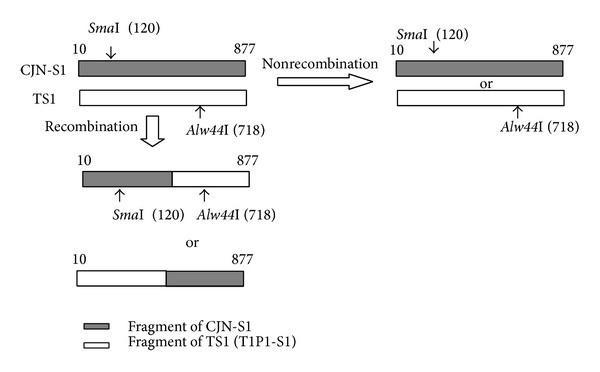
The schematic sketch designed to identify RNA recombination between viral strains. A fragment (868 bp) comprised of the C/preM junction (nt 10~877) of viral RNA extracted from coinfected BHK-21 or C6/36 cells was amplified, cloned, and then used for an RFLP analysis with* Sma*I or* Alw44*I. Two and one recombinant form(s) were, respectively, identified in selected samples from BHK-21 and C6/36 cells, when they were coinfected with the T1P1-S1 and CJN-S1 strains of the Japanese encephalitis virus.

Viral RNA extracted from single infectious centers (ICs) which were randomly selected and picked out from infected BHK-21 or C6/36 cells was subjected to an* Rsa*I RFLP assay as described in our previous report. The result reveals that different strains of the JEV can coinfect a single BHK-21 or C6/36 cell. The C/preM junction comprising 868 nucleotides (nt 10~877) of viral RNA extracted from BHK-21 or C6/36 cells coinfected with the TaP1-S1 and CJN-S1 strains was cloned and used for the* Sma*I-*Alw44*I RFLP analysis (Figure 1). The recombinant forms of the viral genome were actually identified in BHK-21 and C6/36 cells, when they were coinfected by the 2 strains of the JEV. Totally, 20 recombination clones (20.4%) were found from 98 clones coinoculated with the 2 strains in BHK-21 cells while being 5 out 38 (13.1%) in C6/36 cells (Table 1). Probability of occurring RNA recombination was significantly different, compared with the mixed RNA control, in BHK-21 cells while being nonsignificant in C6/36 cells (Table 1). In other words, the frequency of RNA recombination is significantly higher in BHK-21 cells than in C6/36 cells.

### 3.2. Recombination between Genomic RNA and a Transfected RNA Fragment of the Virus

**Figure 2:**
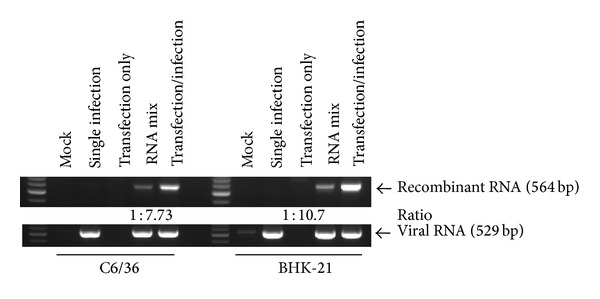
RNA recombination between genomic RNA and a transfected RNA sequence. No fragment was seen in tests with RNA extracted from cells following mock treatment (with neither infection nor transfection), virus infection only, or transfection only. Although amplification of a 564 bp fragment showing RNA recombination was present in the control group which contained a mixture of RNAs extracted from infected and transfected cells, RNA recombination was significantly elevated in BHK-21 and C6/36 cells infected by the Japanese encephalitis virus (Nakayama strain) following transfection with the (+)5′3′-UTR-I plasmid RNA. According to the image-density analysis, it seems that RNA recombination occurred less frequently in mosquito cells. A specific fragment of viral RNA (529 bp) was used as an internal control in all groups with viral infection.

A 564 bp fragment was significantly amplified in BHK-21 and C6/36 cells which were infected by the JEV (Nakayama strain) following transfection with the (+)5′3′-UTR-I RNA plasmid, although a light band was also shown in the control group that contained a mixture of RNAs extracted from transfected cells.A specific fragment of viral RNA (529 bp) was amplified as an internal control in all groups with viral infection. In addition, no fragment presenting an artifact of RNA recombination was shown in the control groups of mock treatment (neither infection nor transfection), transfection with only the (+)5′3′-UTR RNA-I plasmid, or infection with only a single strain. An image-density analysis revealed recombination in BHK-21 cells to be 10.7-fold higher than that of the control group, while it was 7.73-fold higher in C6/36 cells, suggesting that RNA recombination may occur in both mammalian and mosquito cells. However, a slightly lower frequency of RNA recombination was eventually shown in mosquito cells (Figure 2).

### 3.3. Enzymatic Effect on RNA Stability Modulates RNA Recombination between Genomic RNA and a Transfected RNA Fragment of the Virus

**Figure 3:**
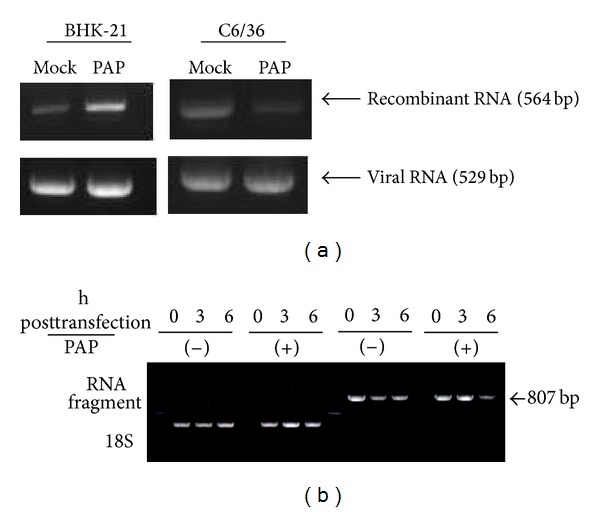
Status of RNA recombination after inhibition by exoribonuclease with PAP (3′-phosphoadenosine-5′-phosphate, an inhibitor of exoribonuclease). (a) The RNA recombination rate increased to a higher level in BHK-21 cells after treatment with PAP, compared to that of untreated cells. In contrast, no effect of PAP on increasing RNA recombination of the virus was shown in C6/36 cells despite a very low level of RNA recombination still being observed. Viral RNA was not affected after treatment with PAP, suggesting exoribonuclease-mediated degradation of transfected RNA fragments might increase RNA recombination of the virus strains, especially in mammalian cells. (b) Treatment with PAP in C6/36 cells did not cause degradation of the transfected (+) RNA fragment at 3 h until 6 h after transfection at which a partial effect appeared.

The RNA recombination rate was shown to have increased to a higher level in BHK-21 cells treated with PAP, the inhibitor of exoribonuclease, compared to untreated cells. In contrast, no effect of PAP on increasing RNA recombination was seen in C6/36 cells; only a low level of RNA recombination was found in this test (Figure 3(a)). Looking at transfected viral fragment (+) RNA in C6/36 cells treated with PAP, enzymatic cleavage by exoribonuclease did not occur at 3 h after transfection while it evidently decreased preservation of such RNA fragment at 6 h after transfection in mosquito cells (Figure 3(b)). Viral genomic RNA was not affected when treated with PAP in both cell types (Figure 3(a)), implying that the transfected viral RNA fragment may not be further degraded by exoribonuclease mostly in mammalian cells; which leads to a higher possibility of occurring RNA recombination in such cells.

### 3.4. Assessment to the Enzymatic Effect on RNA Recombination in Mosquito Cells with Coinfection by Two Different Virus Strains

When we coinfected T1P1-S1 and CJN-S1 strains of JEV into C6/36 cells and treated with PAP, only 1 of 30 clones occurred RNA recombination while 4 out of 31 clones occurred in the control group (without treatment with PAP). The RNA recombination rate did not change significantly (*P* value = 0.370; Yates' chi-square test) in coinfected C6/36 cells and even their function of exoribonuclease was inhibited and thus unable to dissolve viral RNA (Table 2). The result implicated that the low level of viral RNA at the early phase of infection may not be fully exoribonuclease-mediated but, as above, is probably contributed by the RNAi-dependent effect.

### 3.5. Fate of Transfected dsRNA Fragments in Mosquito Cells

**Figure 4:**
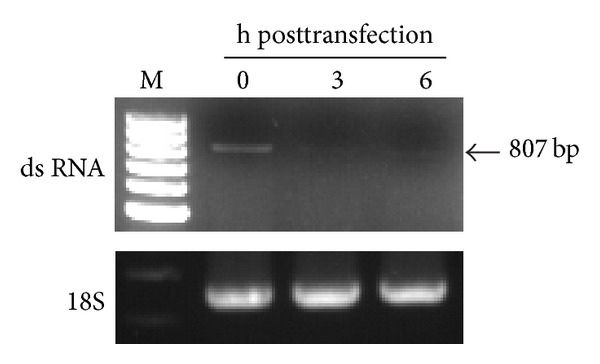
Degradation of double-stranded (ds) RNA fragments transfected into mosquito cells. A fragment (807 bp) of viral RNA extracted from C6/36 cells was detected through an RT-PCR at 0 h after transfection (hpt) with dsRNA derived from (+) or (−) 5′3′-UTR RNA. The transfected dsRNA had faded at 3 and 6 h after transfection, suggesting that dsRNAs may have been cleaved, and thus generated undetectable short interfering RNAs.

The dsRNA intermediates are generally formed during virus replication in host cells, however, which may be cleaved in invertebrate cells. Through an RT-PCR, a corresponding segment of RNA (807 bp) was detected in C6/36 cells immediately after transfection (0 hpt) with a fragment of dsRNA derived from (+) or (−) 5′3′-UTR RNA; however, it had faded by 3 and 6 hpt (Figure 4). This suggests that transfected dsRNAs may have been cleaved and presumably generated short interfering (si)RNAs which were not shown on the gel. It suggested that a part of viral RNAs may be degraded at the early phase of infection, likely to modulate virus growth, in mosquito cells.

### 3.6. Differential RNA Accumulation of Japanese Encephalitis Virus during Early Infection

**Figure 5:**
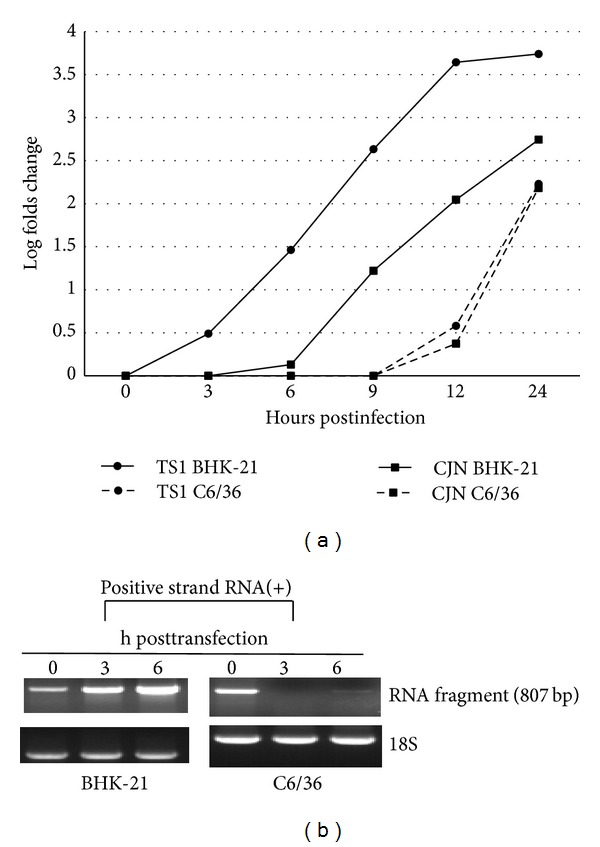
Viral RNA, either T1P1-S1 or CJN-S1, accumulated in C6/36 cells more slowly than in BHK-21 cells. (a) The RNA amount of T1P1-S1 remained at the baseline level until 12 h after infection (hpi) (3.81-fold change), which increased to 169.72-fold at 24 hpi in C6/36 cells. In contrast, T1P1-S1 RNA, respectively, increased to 3.09-, 28.99-, 429.05-, 4396.07-, and 5487.75-fold, at 3, 6, 9, 12, and 24 hpi in BHK-21 cells. The RNA amount of CJN-S1 also accumulated more slowly in C6/36 cells than BHK-21 cells, which remained at the baseline level until 12 hpi (2.36-fold increase) and had increased to 152.32-fold by 24 hpi in C6/36 cells. Although the amount of CJN-S1 RNA did not evidently increase until 6 hpi (1.35-fold change), it increased to 16.64-, 111.43-, and 554.87-fold at 9, 12, and 24 hpi, respectively, in BHK-21 cells. (b) Stability of viral RNA was evaluated after a fragment of (+)5′3′-UTR-II RNA was transfected into BHK-21 or C6/36 cells. Transfected fragments were insignificantly degraded even at 3 or 6 h after transfection in BHK-21 cells while more obvious degradation appeared in C6/36 cells.

Appropriate accumulation of viral RNA in host cells is essential for prosperous production of progeny virions. According to the results, RNAs of both the T1P1-S1 and CJN-S1 strains accumulated more slowly in C6/36 cells than BHK-21 cells (Figure 5(a)). Specifically, the RNA amount of the T1P1-S1 strain remained at the baseline level until 12 hpi (3.81-fold change), compared with an increase of 169.72-fold at 24 hpi in C6/36 cells. In contrast, T1P1-S1 RNA, respectively, increased to 3.09-, 28.99-, 429.05-, 4396.07-, and 5487.75-fold, at 3, 6, 9, 12, and 24 hpi in BHK-21 cells. Similarly, the RNA amount of CJN-S1 also accumulated more slowly in C6/36 cells than in BHK-21 cells. The RNA amount remained at the baseline level until 12 hpi (2.36-fold increase) and subsequently increased to 152.32-fold at 24 hpi in C6/36 cells. In contrast, the RNA amount of CJN-S1 RNA, respectively, increased by 16.64-, 111.43-, and 554.87-fold at 9, 12, and 24 hpi, despite it having been unchanged at 6 hpi (1.35-fold change) in BHK-21 cells. The result revealed that progeny RNA of the virus is delayed to accumulate in mosquito compared to mammalian cells, especially at the early phase of infection. The stability of viral RNA is crucial for the productivity of the progeny virions, which was evaluated after transfection of an RNA fragment prepared from (+)5′3′-UTR-II into either BHK-21 or C6/36 cells. Results showed that transfected fragments had not significantly degraded even at 3 or 6 hpt in BHK-21 cells, while those in C6/36 cells had more obviously degraded (Figure 5(b)), implying that different outcomes of RNA existed in the 2 cell types especially in the early phase of infection.

## 4. Discussion

RNA viruses generate new genetic strains with approximately 6 orders of magnitude higher rates of nucleotide substitutions compared to DNA viruses [28]. Thus, the rate of spontaneous mutations is a critical parameter modeling the genetic structure of viral populations [29]. The primary variation following a mutation may provide for further evolutionary processes, for example, selection and/or recombination [30]. Those in turn lead to the generation of viral strains which are more adept and fit in nature. RNA recombination is now believed to be a strategy for the evolution of many viruses [12], for instance, the poliovirus [31], hepatitis C virus [32], hepatitis D virus [33], and norovirus [34]. A variety of flaviviruses including dengue virus and JEV were also reported to carry out RNA recombination according to bioinformatics inferences [17, 35] and experimental demonstration [25].

Currently, 2 possible mechanisms are reported to lead to the occurrence of recombination [36]: a copy-choice mechanism and a breakage and rejoining mechanism. Of these, the former apparently occurs more commonly as it has been shown in the poliovirus [37], coronaviruses [38], and plant viruses [39]. This mechanism of viral RNA recombinations can further be divided into 3 types: precisely homologous, imprecisely (aberrantly) homologous, and nonhomologous [16]. Among these, precisely homologous recombination through a template-switching (copy-choice) mechanism is probably most common [40]. As in our previous report, different strains of the JEV can coinfect host cells derived from mosquitoes or mammals [25], which actually generates recombinant forms of the virus [30].

In this study, we infected host cells with Nakayama strains of the JEV, followed by transfection of the (+)5′3′-UTR-I RNA fragment. The result was parallel to our previous observation [25], showing imbalanced RNA recombination between BHK-21 and C6/36 cells. Looking at RNA accumulation of JEV in host cells, it takes longer, at least a 24 h difference, in mosquito cells to reach the level of that in mammalian cells. The stability of viral RNA was also shown by degradation of transfected single-stranded RNA fragments, either positive or negative sense, particularly in mosquito cells. It implicated that viral RNA is less stable at least at the early phase of infection by JEV in mosquito cells, which may result in delayed growth of the virus. Since transfected RNA fragments were degraded in both C6/36 cells and BHK-21 cells, RNase cleavage may be actually involved in viral RNA degradation to form small RNAs [41]. However, degradation of transfected RNA fragments in C6/36 cells was partially ameliorated by treatment with PAP, suggesting that viral RNAs are not completely degraded by the RNase cleavage pathway [42]. Perhaps RNA interference (RNAi) plays an important role in the related events [43].

Generally double-stranded replicative-form RNA (dsRF-RNA) accumulates to provide an immediate signal which activates specific transcription factors such as type-I interferon (IFN) [44] and facilitates the triggering of intracellular innate immunity in mammalian cells [45]. On the other hand, dsRNAs formed in invertebrate cells are usually cleaved to be siRNA that consequently degrades viral RNAs [46], leading to RNAi-mediated innate immunity [47]. Small RNAs ranging from 10 to 24 mer have been identified in C6/36 cells infected by West Nile virus [43]. We have also detected normal expression of Dicer-2 in C6/36 cells infected by JEV for 12 h although it was almost half of inhibition at 6 hpi (data not shown). As a result, dsRF-RNA of the JEV may have a great potential for viral RNA degradation at least in the early phase of infection in mosquito cells. This adjustment of RNA amount is believed to be the way for a delay in RNA accumulation and thus a lower frequency of RNA recombination of the JEV. In contrast, dsRNAs are recognized as a central component of IFN and therefore are incapable of mediating RNAi in mammalian cells [48]. Eventually, our results have shown that protection of RNA from RNase cleavage increases the efficiency of RNA recombination particularly in mammalian cells.

RNA recombination creates advantageous genotypes by evolutionary jumps [30], which permits the removal of deleterious genes based on the notion of “Muller's ratchet” from the host cell, usually mammalian cells [12]. Notably, viral RNAs usually accumulated at a lower amount in mosquito cells through RNase cleavage as well as RNA-mediated pathways, leading to stagnancy of RNA recombination which may brake evolution of the JEV and probably most, if not all, arboviruses which are maintained in nature by alternate cycles involving mosquitoes and vertebrates [28].

## Figures and Tables

**Table 1:** Identification of RNA recombination of the Japanese encephalitis virus based on a fragment (868 bp) comprised of the C/preM junction (nt 10~877) of viral RNA extracted from coinfected BHK-21 or C6/36 cells using an RFLP analysis with restriction enzymes *SmaI* or *Alw44I*.

Treatment	BHK-21 cells		C6/36 cells	
	Number of detection	Number of recombination	Number of detection	Number of recombination
Coinfected viral genomic RNA	98	20 (20.4%)	38	5 (13.1%)
Mixed RNA∗	44	2 (4.5%)	39	3 (7.7%)
Stastistical analysis∗∗	*P* < 0.05		*P* > 0.05	

*Mixed RNA was a mixture of RNAs separately extracted from T1P1-S1 and CJN-S1 strains of the Japanese encephalitis virus, being used as the internal control.

**Yates' chi-square test was used to assess the difference of RNA recombination in cells coinfected by two virus strains at 5% level of significance.

**Table 2:** Detection of RNA recombination in C6/36 cells simultaneously infected by T1P1-S1 and CJN-S1 in the presence of PAP (3′-phosphoadenosine-5′-phosphate, an inhibitor of exoribonuclease).

Recombination	PAP treatment		
	−	+	Total
+	4	1	6
−	27	29	85
Total	**31**	**30**	**91**

Yates' chi-square test was used to assess the difference of RNA recombination (*P* value = 0.370).
